# Brachial and central blood pressure and arterial stiffness in adult elite athletes

**DOI:** 10.1007/s00421-021-04662-z

**Published:** 2021-03-13

**Authors:** Fabian Tomschi, Hannah Ottmann, Wilhelm Bloch, Marijke Grau, Hans-Georg Predel

**Affiliations:** 1 Institute of Cardiology and Sports Medicine, German Sport University Cologne, Am Sportpark Müngersdorf 6, 50933 Cologne, Germany; 2 Molecular and Cellular Sports Medicine, German Sport University Cologne, Am Sportpark Müngersdorf 6, 50933 Cologne, Germany; 3 The German Research Center of Elite Sport (Momentum), German Sport University Cologne, Am Sportpark Müngersdorf 6, 50933 Cologne, Germany

**Keywords:** Blood pressure, Pulse wave velocity, Arterial stiffness, Reference values, Professional sport

## Abstract

**Purpose:**

Measures of arterial stiffness (AS) and central blood pressure (BP) are indicators for cardiovascular health and possess a high prognostic value in the prediction of cardiovascular events. The effects of physical training are widely unexplored in the context of competitive, high-performance sports. Therefore, we aimed to present possible reference values of brachial and central BP and of AS of adult elite athletes compared to a control group.

**Methods:**

A total of 189 subjects participated in this cross-sectional study. Of these were 139 adult elite athletes (70 male, 69 female) performing on top-national and international level, and 50 control subjects (26 male, 24 female). Resting brachial and central BP and aortic pulse wave velocity (PWV) were measured and were compared in terms of sex, sport category, and age of the athletes.

**Results:**

Results show no difference between athletes and controls in any parameter. Women exhibit lower brachial and central BP and AS values compared to men. PWV is positively correlated with age. Evaluation of the parameters according to the different sport categories showed that endurance athletes exhibit lower BP and PWV compared to other athletes.

**Conclusions:**

This study presents brachial and central BP and PWV values of athletes, suggesting that high-performance sport does not negatively impact AS. The proposed reference values might support a more detailed evaluation of elite athlete’s cardiovascular and hemodynamic system and a better assignment to possible risk groups.

## Introduction

It is well known that physical activity results in various health benefits, especially on the cardiovascular (CV) system (Blair 1989). Hence, in elite athletes, the CV risk can be described as very low (Bruno et al. 2011) and it can be assumed that the vascular ageing process is also relatively slow due to several factors. These include, among others, that elite athletes very seldom smoke (Peretti-Watel et al. 2003) and above all are highly active. In the general population, regular physical activity has a well-known blood pressure (BP) lowering effect (Chobanian et al. 2003). However, hypertension is the most common cardiovascular condition in athletes (Caselli et al. 2017; Pelliccia et al. 2017; Matos et al. 2011). The underlying reasons are not fully clarified but it was suggested that the mental stress associated with regular competition at top level and the regular high training amount of more than 14 h per week (Berge et al. 2015) might increase BP. Further, brachial BP might be increased due to the phenomenon of the ‘spurious systolic hypertension’ (SSH) (Mahmud and Feely 2003) mainly due to extreme pulse pressure (PP) amplification. In this case, central aortic BP can be 30–40 mmHg less that the brachial BP. This phenomenon was mainly observed in young, tall and very active young men (O'Rourke et al. 2000). It was, therefore, recommended to measure central BP and the aortic pressure wave in subjects who exhibit SSH because a normal central systolic and diastolic BP and a normal aortic pressure waveform most likely indicate that these subjects are not at increased cardiovascular risk (Mahmud and Feely 2003). Besides, athletes might make extensive use of ‘ergogenic aids’ such as a disproportionately high consumption of caffeine, prescription medications or dietary supplements that might increase BP (Leddy and Izzo 2009).

It was demonstrated that the measurements of central BP and aortic pulse wave velocity (PWV), a direct measure of arterial stiffness (AS), are better related to future cardiovascular events compared to the conventional brachial BP measurement (Karras et al. 2012; McEniery et al. 2014). Both, central BP and PWV were independently correlated with organ damage and cardiovascular manifestations (Kotsis et al. 2011).

However, to the best of our knowledge, there are no scientific publications presenting possible reference values of central BP and PWV of adult elite athletes. Due to the fact that these athletes have a highly active lifestyle, common reference values might be problematic to obtain when referring to adult elite athletes.

Therefore, the aim of the study was to present possible reference data of hemodynamic values, such as brachial and central BP and PWV, of adult elite athletes which can serve as a basis for clinical evaluation. The knowledge gained by this study might be of importance for clinicians dealing with adult elite athletes as the data presented in this study possess an additional clinical and prognostic value for more detailed risk stratification in adult elite athletes.

## Methods

### Ethics

The study and the used protocols were approved by the ethics committee of the German Sport University Cologne. These protocols are in line with the Declaration of Helsinki. Participants gave written informed consent to participate in the study.

### Participants

The inclusion criterion for participation in this study was to have an active status within the elite A, B, and C squad system of the German Olympic Federation. This squad system defines the performance level of the athlete. Depending on the discipline, A-level athletes have achieved top-level positions at Olympic Games or World Championships, B-level athletes displayed considerable performance development and are prospective candidates for A-level status, and C-level included the highest national level for prospective young athletes who exhibit the potential to perform at the top international level or who competed successfully in international junior competitions (Zinner et al. 2015). All athletes were selected for participation by their respective sports federation, so they can be considered to be among the national elite.

One hundred and seventy-nine participants (age 23.3 ± 3.7, range 18–36 years) were recruited in total. Of these, 139 were adult elite athletes (70 male, 69 female). Anthropometric data of athletes were as follows: 23.3 ± 3.8 years, 177.9 ± 9.4 cm, 72.1 ± 12.8 kg, 22.6 ± 2.5 kg/m^2^. 29 different sports were represented by the participating athletes. Due to varying samples sizes, the athletes were clustered into the following sport groups as done before (Koehler et al. 2012; Zinner et al. 2015; Tomschi et al. 2018): endurance sports (*n* = 23) [triathlon (*n* = 1), long distance running (*n* = 12), middle distance running (*n* = 9), heptathlon (*n* = 1)]; return and team sports (*n* = 34) [badminton (*n* = 1), baseball (*n* = 8), basketball (*n* = 1), ice hockey (*n* = 1), soccer (*n* = 9), handball (*n* = 7), tennis (*n* = 1), table tennis (*n* = 4),]; combat sports (*n* = 28) [boxing (*n* = 2), judo (*n* = 17), karate (*n* = 2), wrestler (*n* = 5), taekwondo (*n* = 2)]; aesthetic and individual sports (*n* = 24) [fencing (*n* = 18), horse riding (*n* = 2), dancing (*n* = 2), race driver (*n* = 1)]; and strength and power sports (*n* = 30) [sprint (*n* = 8), bob-sledge (*n* = 1), high-jump (n = 1), canoe (*n* = 2), lifesaver swimming (*n* = 1), rowing (*n* = 1), pole-vault (*n* = 13), and javelin (*n* = 1)].

Furthermore, 50 participants were recruited to serve as a control group (26 male, 24 female) and anthropometric data were as follows: 23.0 ± 3.0 years, 175.3 ± 8.8 cm, 71.6 ± 11.3 kg, 23.2 ± 2.5 kg/m^2^. These control participants were moderately active university students and reported no more than 4 h of physical activity per week. These control participants did not exert any performance orientated training.

Both groups of participants were told not to consume caffeine or alcohol and refrain from highly vigorous physical activity for at least 12 h prior to the data collection. The time of the menstrual cycle was not registered in female subjects. Anthropometric data of male and female athletes and of athletes of the different sport types are presented in Table 1.

**Table 1:** Anthropometric and hemodynamic data of male and female athletes and of male and female controls

	Overall male (*n* = 96)	Overall female (*n* = 93)	Athletes (*n* = 139)	Male athletes (*n* = 70)	Female athletes (*n* = 69)	Controls (*n* = 50)	Male controls (*n* = 26)	Female controls (*n* = 24)
Age [years]	23.2 ± 3.6 (22.5–24.0)	23.4 ± 3.7 (22.5–24.1)	23.3 ± 3.9 (22.7–24.0)	23.2 ± 3.7 (22.3–24.0)	23.5 ± 4.1 (22.5–24.5)	23.0 ± 3.0 (22.1–23.9)	23.4 ± 3.5 (22.0–24.8)	22.5 ± 2.4 (21.5–23.6)
Height [cm]	183.1 ± 8.1 (181.5–184.7)	171.1 ± 6.0 (169.9–172.3)	177.9 ± 9.4 (176.3–179.5)	183.7 ± 8.6 (181.6–185.7)	172.0 ± 6.1 (172.0–173.5)	175.3 ± 8.8 (172.8–177.7)	181.6 ± 6.6 (178.9–184.3)	168.4 ± 4.7 (166.4–170.4)
Weight [kg]	79.8 11.3 (77.5–82.1)	64.0 ± 11.3 (77.5–82.1)	72.1 ± 12.8 (70.0–74.3)	79.7 ± 12.4 (76.8–82.7)	64.4 ± 7.7 (62.6–66.3)	71.6 ± 11.3 (68.4–74.9)	80.0 ± 8.1 (76.7–83.2)	62.6 ± 6.4 (59.9–65.3)
BMI [kg/m^2^]	23.6 ± 2.6 (23.7–24.1)	21.8 ± 2.1 (21.4–22.3)	22.6 ± 2.5 (22.1–23.0)	23.4 ± 2.7 (22.7–24.0)	21.7 ± 2.1 (21.2–22.2)	23.2 ± 2.5 (22.5–23.9)	24.2 ± 2.3 (23.3–25.2)	22.1 ± 2.1 (21.2–22.9)
bSysBP [mmHg]	128.4 ± 11.5 (126.1–130.7)	115.8 ± 9.4 (113.9–117.8)*	122.2 ± 12.2 (120.2–124.3)	128.2 ± 11.9 (125.4–131.1)	116.2 ± 9.2 (114.0–118.4)	122.1 ± 12.5 (118.6–125.7)	128.9 ± 10.5 (124.7–133.1)	114.8 ± 10.3 (110.4–119.1)
bDiaBP [mmHg]	76.3 ± 7.5 (74.8–77.8)	73.1 ± 8.3 (71.4–74.8)*	74.4 ± 8.0 (73.1–75.8)	75.8 ± 7.2 (74.1–77.6)	73.0 ± 8.5 (71.0–75.1)	75.6 ± 8.2 (73.3–78.8)	77.7 ± 8.2 (74.3–81.0)	73.4 ± 7.8 (70.1–76.7)
cSysBP [mmHg]	112.3 ± 8.5 (110.5–114.0)	103.2 ± 9.3 (101.2–105.1)*	107.1 ± 10.2 (105.4–108.8)	111.3 ± 8.9 (109.2–113.5)	102.8 ± 9.6 (100.5–105.1)	109.6 ± 9.3 (106.9–112.2)	114.7 ± 6.7 (112.0–117.4)	104.0 ± 8.7 (100.4–107.7)
cDiaBP [mmHg]	78.1 ± 7.55 (76.6–79.6)	74.6 ± 8.2 (72.9–76.3)*	76.1 ± 7.9 (74.8–77.4)	78.1 ± 7.6 (76.0–79.4)	74.6 ± 8.2 (72.5–76.5)	77.1 ± 8.5 (74.6–79.5)	79.0 ± 8.7 (75.5–82.5)	74.9 ± 7.9 (71.9–78.3)
PWV [m/s]	5.3 ± 0.4 (5.2–5.3)	4.9 ± 0.4 (4.8–5.0)*	5.1 ± 0.5 (5.0–5.2)	5.4 ± 0.5 (5.1–5.4)	4.9 ± 0.4 (4.8–5.0)	5.1 ± 0.4 (5.0–5.2)	5.4 ± 0.4 (5.2–5.4)	4.8 ± 0.4 (4.7–4.9)

Data are presented as means ± standard deviation (95% CI)

*BMI* body mass index, b*SysBP* brachial systolic blood pressure, *bDiaBp* brachial diastolic blood pressure, *cSysBP* central systolic blood pressure, *cDiaBP* central diastolic blood pressure, *PWV* pulse wave velocity

^*^P < 0.05 vs. overall male

### Measurements

All measurements were performed using a Mobil-O-Graph device (IEM, Stolberg, Germany) as a validated device for the measurement of brachial BP (Franssen and Imholz 2010), central BP (Weiss et al. 2012), and PWV (Hametner et al. 2013) with a novel transfer function-like algorithm, using brachial cuff-based waveform recordings. For central systolic pressure, calculation and other measures of AS an integrated transfer function (ARCSolver algorithm) was used (Weber et al. 2011; Wassertheurer et al. 2010). This algorithm published by Wassertheurer et al. (2010) is based on the algorithm developed by Karamanoglu et al. (1993). Wassertheurer et al. (2010) used a modified cuff by adding a high-fidelity pressure sensor with increased accuracy which allowed them to transform the brachial artery pressures to aortic pressures, using a frequency-based general transfer function similar to a previous one developed by Karamanoglu et al. (1993). Indeed, the mean bias between the ARCSolver method, as it is known, and the technique of Karamanoglu et al. (1993) was 0.1 mmHg (SD 3.1 mmHg).

First, the device measures the peripheral BP. After 30 s of pause, the device measures further hemodynamic parameters which are presented in the software output. The time duration of the entire measurement is 3–4 min. The parameters that were evaluated were systolic (bSysBP) and diastolic brachial BP (bDiaBP), systolic (cSysBP) and diastolic central BP (cDiaBP) and PWV. PWV was shown to be the most reliable measure of AS (O'Rourke et al. 2002; Kim et al. 2007). Compared to the invasive measurement of the central systolic pressure and established radial tonometry with inbuilt generalized transfer function, the ARCSolver algorithm showed good agreement (Weber et al. 2011; Wassertheurer et al. 2010).

**Fig. 1:**
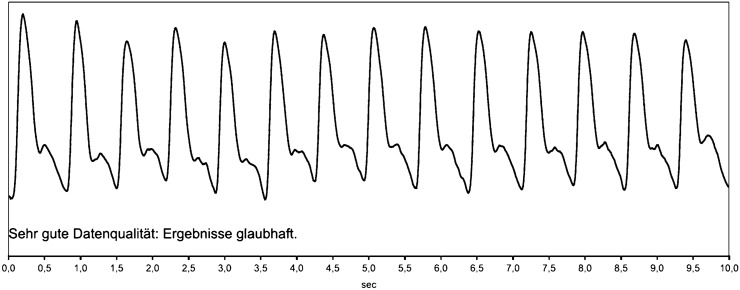
Representative pulse pressure wave using the Mobil-O-Graph device (IEM Gmbh, Stolberg, Germany) using an oscillometric cuff. Sehr gut Datenqualität: Ergebnisse glaubhaft = Very good data quality: Results plausible (translated from German)

All hemodynamic measurements were conducted following the guidelines for the management of arterial hypertension (Williams et al. 2018). Participants were in a sitting position (after 10 min of rest) in a quiet room at the same time of day (between 9 and 11 a.m.). The cuff was placed at the level of the heart with cuffs of correct size and in accordance to the participants’ arm circumference. Measurements were repeated, if the quality of the measured arterial pressure wave was poor. By doing so, it was possible obtain an adequate good quality arterial pressure wave from every participating subject. The quality of the arterial pressure wave was checked automatically by the software program and visually by trained medical staff. A representative pressure wave can be found in Fig. 1.

### Statistical analyses

Statistical analyses of the data were performed using statistics software package GraphPad Prism 6 (La Jolla, USA). The data are reported as means ± standard deviation (SD) and 95% confidence interval (CI).

To compare hemodynamic values (bSysBP, bDiaBP, cSysBP, cDiaBP, and PWV) between athletes and controls, a two-way ANOVA with the factors ‘gender’ (male or female) and ‘group’ (control or athlete) was applied. Besides, a two-way ANOVA with the factors ‘gender’ (male or female) and ‘sport category’ (endurance, return and team, combat, aesthetic and individual, strength and power) was applied to determine differences in hemodynamic parameters between the five different sport categories and between male and female athletes. If main effects were observed, Tukey post hoc analyses were used to locate differences between sport categories. Bonferroni post hoc tests were performed to reveal significant differences in interactions between ‘gender’ and ‘sport category’ or ‘gender’ and ‘group’, respectively.

To evaluate the effect of age on hemodynamic parameters, Pearson’s correlation analyses and regression analyses were conducted.

## Results

Means, SD and 95% CI of the measured hemodynamic parameters of all subjects (athletes and controls) are presented in Table 1. Overall, no main effect for the factor group (athlete or control) was observed between athletes compared to controls in bSysBP, bDiaBP, cSysBP, cDiaBP, and PWV, respectively. A main effect was observed for the factor gender. Female subjects showed significantly lower values in bSysBP (*P* < 0.001), bDiaBP (*P* = 0.008), cSysBP (*P* < 0.001), cDiaBP (*P* = 0.005) and PWV (*P* < 0.001) compared to male subjects. No group x gender interaction was observed for any parameter.

Means, SD and 95% CI of the measured hemodynamic parameters of all athletes separated by sport category are presented in Table 2. Statistical analyses of differences between sport categories revealed that bSysBP was significantly different between sport categories (*P* = 0.005). Post hoc analysis revealed that endurance athletes showed lower bSysBP values compared to strength and power (*P* = 0.040), combat (*P* = 0.009) and team and racket athletes (*P* = 0.017). bDiaBP was not different between the different sport categories.

**Table 2:** Anthropometric and hemodynamic data of athletes separated by sport category

Sport category	Endurance (*n* = 23)	Strength and power (*n* = 30)	Combat (*n* = 28)	Team and racket (*n* = 34)	Individual and aesthetic (*n* = 24)
Age [years]	23.0 ± 2.2 (22.0–23.1)	24.7 ± 5.0 (22.8–26.6)	22.4 ± 3.0 (21.2–23.5)	23.1 ± 4.1 (21.7–24.5)	23.4 ± 3.9 (21.8–25.0)
Height [cm]	175.0 ± 7.2 (171.9–178.1)	180.6 ± 9.8 (177.0–184.3)	174.7 ± 7.3 (171.9–177.5)	178.0 ± 11.4 (174.1–182.0)	180.8 ± 8.8 (177.1–184.4)
Weight [kg]	61.7 ± 7.3 (58.6–64.9)	75.2 ± 11.4 (71.0–79.5)	71.9 ± 10.4 (67.9–75.9)	76.1 ± 15.4 (70.7–81.4)	73.0 ± 12.7 (67.7–78.4)
BMI [kg/m^2^]	20.1 ± 1.4 (19.5–20.7)	22.9 ± 2.2 (22.1–23.7)	23.4 ± 2.1 (22.6–24.2)	23.4 ± 2.9 (22.4–24.4)	22.3 ± 12.7 (21.4–23.1)
bSysBP [mmHg]	115 ± 9 (111–119)	123 ± 13 (119–128)*	125 ± 12 (120–130)*	124 ± 14 (119–129)*	122 ± 11 (117–127)
bDiaBP [mmHg]	72. ± 6. (69–75)	73 ± 8 (70–76)	76 ± 76 (73–79)	76 ± 7 (73–78)	75 ± 10 (71–79)
cSysBP [mmHg]	101 ± 8 (97–104)	107 ± 10 (103–111)	109 ± 9 (106–113)*	109 ± 11 (106–11)*	108 ± 11 (104–113)*
cDiaBP [mmHg]	74 ± 6 (71–77)	75 ± 8 (72–78)	78 ± 8 (75–81)	77 ± 7 (75–80)	76 ± 9 (72–80)
PWV [m/s]	4.8 ± 0.0 (4.6–4.9)	5.2 ± 0.5 (5.0–5.3)*	5.2 ± 0.5 (5.0–5.4)*	5.1 ± 0.5 (5.0- 5.3)*	5.1 ± 0.4 (5.0–5.3)*

Data are presented as means ± standard deviation (95% CI)

*BMI* body mass index, b*SysBP* brachial systolic blood pressure, *bDiaBp* brachial diastolic blood pressure, *cSysBP* central systolic blood pressure, *cDiaBP* central diastolic blood pressure, *PWV* pulse wave velocity

^*^P < 0.05 vs. endurance

cSysBP was significantly different between the sport categories (*P* < 0.001). Post hoc analysis revealed that cSysBP was significantly lower in endurance athletes compared to combat (*P* = 0.004), team and racket (*P* = 0.002) and individual and aesthetic athletes (*P* = 0.018). cDiaBP was not different between the sport categories.

Statistical analyses revealed that PWV was significantly different between the sport categories (*P* = 0.002). Post hoc analysis revealed that PWV was significantly lower in endurance athletes compared to the categories strength and power (*P* = 0.005), combat (*P* = 0.006), team and racket (*P* = 0.009) and individual and aesthetic (*P* = 0.015).

**Fig. 2:**
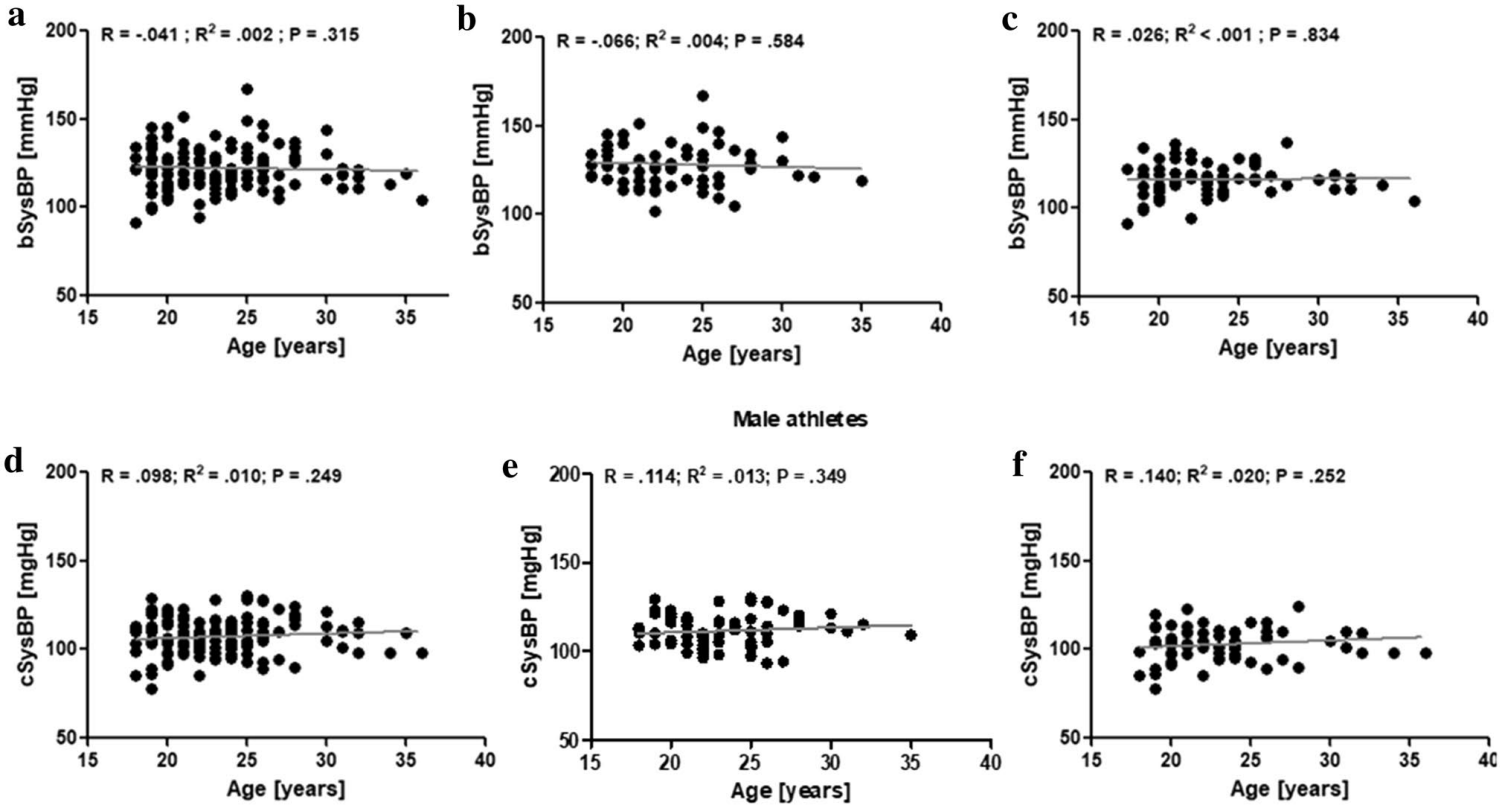
Correlation analyses of brachial systolic blood pressure (bSysBP) and age of all athletes (**a**), male athletes (**b**), and female athletes (**c**). Correlation analyses of central systolic blood pressure (cSysBP) and age of all athletes (**d**), male athletes (**e**), and female athletes (**f**)

**Fig. 3:**
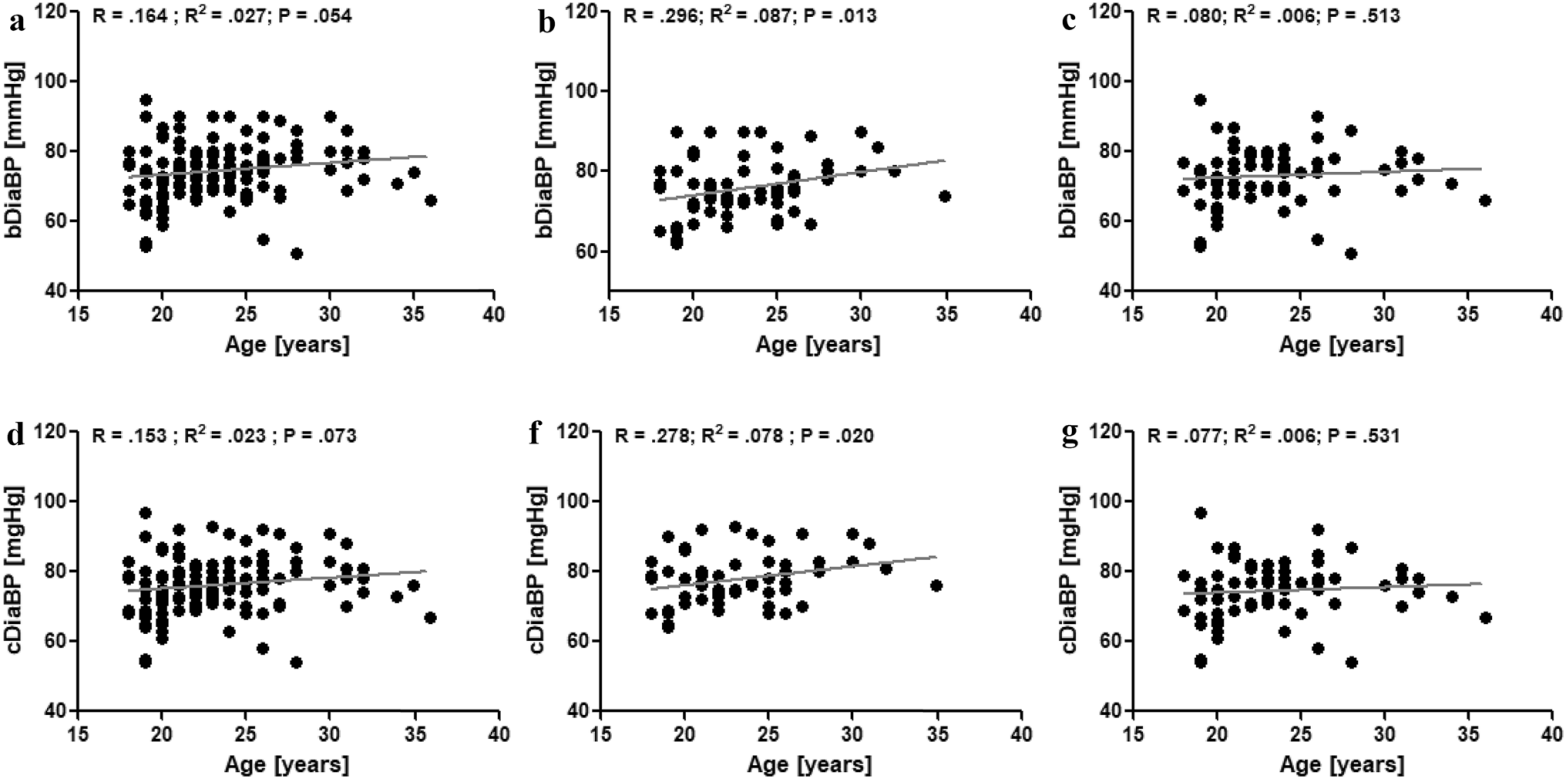
Correlation analyses of brachial diastolic blood pressure (bDiaBP) and age of all athletes (**a**), male athletes (**b**), and female athletes (**c**). Correlation analyses of central diastolic blood pressure (cDiaBP) and age of all athletes (**d**), male athletes (**e**), and female athletes (**f**)

**Fig. 4:**
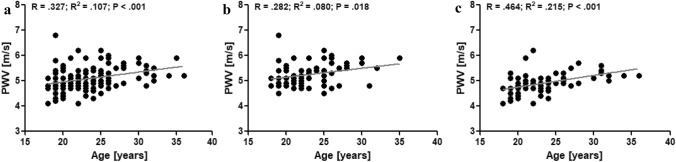
Correlation analyses of aortic pulse wave velocity (PWV) and age of all athletes (**a**), male athletes (**b**), and female athletes (**c**)

In athletes, no correlation was observed between age and bSysBP (Fig. 2a). Besides, no correlation was found in athletes between age and cSysBP (Fid. 2d). This was also observed in male athletes, exclusively (Fig. 2b + e) and in female athletes, exclusively (Fig. 2c + f). In all athletes, age did not correlate with bDiaBP and cDiaBP (Fig. 3a + d), respectively. In male athletes, age positively correlated with bDiaBP (*P* = 0.013; Fig. 3b) and cDiaBP (*P* = 0.020; Fig. 3e), respectively. These correlations were not observed in female athletes (Fig. 3c + f). Correlation analyses revealed that PWV was significantly correlated (*P* < 0.001) to the age of the athletes (Fig. 4a). This was observed for male (*P* = 0.009; Fig. 4b) and female athletes (*P* < 0.001; Fig. 4c), respectively.

## Discussion

To the best of our knowledge, this study first time presents values of central BP and PWV of adult elite athletes performing different sports and comparing those to values of an age-matched healthy control group. The participating athletes of this study exhibit brachial BP values that are in a healthy range. Male athletes are on average in the normal systolic BP range and female athletes are in the optimal systolic BP range with respect to the ESH/ESC guidelines (Williams et al. 2018). Further, PWV values of the participating athletes are also lower when comparing them to possible reference data provided by the European Society of Cardiology (ESC) (The Reference Values for Arterial Stiffness' Collaboration 2010). These reference values show that healthy subjects under the age of 30 have a mean PWV of 6.2 m/s. However, it is important to consider that the methods used are different which might be problematic to compare the results of the present study to the given reference values of the ESC. Due to the fact that the device used in the present study shows great reproducibility (Wassertheurer et al. 2010; Weber et al. 2011), this comparison most likely indicates that the elite athletes exhibit more favourable PWV values compared to the non-athletic population. Further, comparing brachial and central BP values and AS values of elite athletes of the present study to the brachial and central BP and PWV values of the recruited control group, results show that athletes do not exhibit significantly different values in any of the measured parameters. Interestingly, PWV values of the control group are also lower than the above mentioned ESC reference values (The Reference Values for Arterial Stiffness' Collaboration 2010). This is probably due to the mean young age of the control group (23.0 ± 3.0 years). The age of all the participating subjects is of crucial importance in the comparison of PWV values given in the literature as ageing is the main contributor to vascular ageing (Lakatta and Levy 2003). This positive correlation between age and PWV is also observed for athletes in the present study and does not depend on the gender of the athletes. However, the age range of the athletes is rather narrow as athletes can usually only perform top-elite level in a specific age range. This fact limits the informative value (low *R*^2^ values; see Fig. 4a–c) of the correlation analyses provided. Further studies should be encouraged to explore the vascular health of older (former) athletes.

Interestingly, neither bSysBP nor cSysBP are positively correlated to the age of the athletes of the present study. This is also observed for male and female athletes, exclusively. It is generally assumed that PWV and BP show a great positive correlation even in young age as the flexibility of the arteries is manifested in BP values (Cheung 2010). Extensive physical activity, as performed by elite athletes is known to affect BP (Caselli et al. 2017). However, an early impaired vascular function associated with increased AS usually precedes elevations in resting brachial BP (Kim et al. 2015). This might be one reason for the lack of correlation between age and bSysBP or cSysBP, respectively.

Diastolic BP was shown to be of clinical importance as it indicates a potential risk for future cardiovascular complications (Franklin 2007) and the present study shows that bDiaBP and cDiaBP are positively correlated with age in male athletes. Interestingly, this is not observed in female athletes. It is known that diastolic BP rises with age predominantly in the male population in age groups under 45 years (Martins et al. 2001). This might explain this correlation found in male athletes.

The present data show in general that female subjects exhibit a lower bSysBP, cSysBP and a lower PWV. These results are in line with the current literature showing that young women who do not perform professional sports show lower brachial BP as well as lower PWV values (The Reference Values for Arterial Stiffness' Collaboration 2010). In addition, in athletes, it was shown that female athletes exhibit lower brachial BP values compared to male athletes (Caselli et al. 2017). The exact reasons for the lower BP and PWV values observed in pre-menopausal women are not entirely understood but it is suggested that the hormone oestrogen plays a pivotal role in cardiovascular and hemodynamic sex differences. Estrogens influence the vaso-reactivity that has a relaxing effect on peripheral resistance vessels (Li and Kloner 1995; Penna et al. 2009).

Looking at the hemodynamic values of athletes of the different sport categories, it is observed that endurance athletes have lowest bSysBP values. These values are lower than those of the athletes of the categories combat, and team and racket sports. In addition, cSysBP is lowest in endurance athletes and significantly lower compared to cSysBP values of athletes of the categories combat, team and racket, and individual and aesthetic sports. These results are in line with the current knowledge which is that in recreational sport settings endurance dominated exercise programs are particularly effective in lowering peripheral and central systolic BP (Tanaka et al. 2000). This phenomenon was also previously observed in elite athletes. It was shown in a meta-analysis that endurance-trained athletes exhibit lower brachial BP compared to strength-trained athletes (Berge et al. 2015).

In the present study, endurance athletes show significantly lower PWV values compared to all other athletes of other categories. It was demonstrated that rather endurance exercise interventions improve PWV compared to resistance exercise interventions (Ashor et al. 2014). Several studies further suggest that especially strength training interventions can lead to increases in BP and AS (Otsuki et al. 2007; Bertovic et al. 1999; Choi et al. 2010; Collier et al. 2011; Cortez-Cooper et al. 2005). However, studies also report that resistance training interventions do not lead to increases of AS (Casey et al. 2007; Cortez-Cooper et al. 2008; Rakobowchuk et al. 2005). In a meta-analysis, it was reported that high-intensity resistance training leads to increases in AS of 11.6%. In contrast, moderate-intensity resistance training did not show such association (Miyachi 2013). It was further suggested that combined training, i.e. resistance and aerobic endurance training, did not induce stiffening of the arteries (Li et al. 2015). Besides, Montero et al. showed that combined training interventions do not lead to increases in AS but that aerobic endurance interventions are more effective in reducing AS (Montero et al. 2015). In the present study, all sport categories, except for endurance athletes, can be described as hybrid sport categories in which a well-established endurance and strength capacity is a prerequisite for success in the respective sport category (Nader 2006). Differential effects stemming from different training stimuli on the vascular system are, therefore, unlikely and support the suggestion that combined training does not lead to increased AS (Li et al. 2015).

The major strength of the present study is that a high number of adult elite athletes was recruited who perform on top national and international level. However, we also need to acknowledge some limitations. The cross-sectional study design needs to be mentioned as a limitation because it cannot directly measure cause and effect. Yet, all participating athletes were part of the national elite squat system suggesting that a chronic sport type specific training can be assured for every athlete over a long duration of time. Neither specific data of the training history of the elite athletes were obtained, nor were exact training schedules of the athletes recorded. Besides, the effect of the menstrual cycle in female athletes was not considered in this study. Though, studies show that the phase of the menstrual cycle has little effects on hemodynamic values (Augustine et al. 2018; Papaioannou et al. 2009). Further, all athletes and control subjects were in a young adult age. It would be interesting to study the effects of performance orientated training in older adults to evaluate whether intensive training intensifies the age-related physiological processes that contribute to mechanisms, such as earlier vascular ageing. Lastly, it needs to be mentioned that all measurements in the present study were conducted with one and the same device and the presented values are stated as means with SD and 95% CI. Yet, values obtained in the individual athlete or patient might not fit into the presented results for a number of reasons (e.g. different diagnostic device used, different time of the day, different pre-measurement activity, prior use of ‘ergogenic aids’ such as large amounts of caffeine, or a potential lack of accuracy for estimates of central aortic blood pressures and PWV).

## Conclusion

This study for the first time presents possible reference values for brachial and central BP and PWV, a measure of AS. The results indicate that athletes in general do not exhibit different hemodynamic values compared to an age-matched, healthy control group. Within athletes, those who perform endurance dominant sports show lower BP and AS values. The present data suggest that high-performance sport in young athletes does not negatively impact AS.

Furthermore, the proposed reference values not only enable a general evaluation of elite athlete’s cardiovascular and hemodynamic system, but also a better assignment to risk groups. Due to their high prognostic value AS and central BP should be additionally considered to the athlete’s brachial BP.

## Abbreviations

CV: Cardiovascular
BP: Blood pressure
SSH: Spurious systolic hypertension
PP: Pulse pressure
PWV: Aortic pulse wave velocity
AS: Arterial stiffness
SD: Standard deviation
CI: Confidence interval
bSysBP: Brachial systolic blood pressure
bDiaBP: Brachial diastolic blood pressure
cSysBP: Central systolic blood pressure
cDiaBP: Central diastolic blood pressure
